# Modulation of Calcium Transients in Cardiomyocytes by Transient Receptor Potential Canonical 6 Channels

**DOI:** 10.3389/fphys.2020.00044

**Published:** 2020-02-14

**Authors:** Azmi A. Ahmad, Molly E. Streiff, Chris Hunter, Frank B. Sachse

**Affiliations:** ^1^Nora Eccles Harrison Cardiovascular Research and Training Institute, University of Utah, Salt Lake City, UT, United States; ^2^Department of Biomedical Engineering, University of Utah, Salt Lake City, UT, United States

**Keywords:** transient receptor potential canonical channels, transient receptor potential canonical 6, cardiomyocyte, calcium transient, mechanosensitivity

## Abstract

Transient receptor potential canonical 6 (TRPC6) channels are non-selective cation channels that are thought to underlie mechano-modulation of calcium signaling in cardiomyocytes. TRPC6 channels are involved in development of cardiac hypertrophy and related calcineurin-nuclear factor of activated T cells (NFAT) signaling. However, the exact location and roles of TRPC6 channels remain ill-defined in cardiomyocytes. We used an expression system based on neonatal rat ventricular myocytes (NRVMs) to investigate the location of TRPC6 channels and their role in calcium signaling. NRVMs isolated from 1- to 2-day-old animals were cultured and infected with an adenoviral vector to express enhanced-green fluorescent protein (eGFP) or TRPC6-eGFP. After 3 days, NRVMs were fixed, immunolabeled, and imaged with confocal and super-resolution microscopy to determine TRPC6 localization. Cytosolic calcium transients at 0.5 and 1 Hz pacing rates were recorded in NRVMs using indo-1, a ratio-metric calcium dye. Confocal and super-resolution microscopy suggested that TRPC6-eGFP localized to the sarcolemma. NRVMs infected with TRPC6-eGFP exhibited higher diastolic and systolic cytosolic calcium concentration as well as increased sarcoplasmic reticulum (SR) calcium load compared to eGFP infected cells. We applied a computer model comprising sarcolemmal TRPC6 current to explain our experimental findings. Altogether, our studies indicate that TRPC6 channels play a role in sarcolemmal and intracellular calcium signaling in cardiomyocytes. Our findings support the hypothesis that upregulation or activation of TRPC6 channels, e.g., in disease, leads to sustained elevation of the cytosolic calcium concentration, which is thought to activate calcineurin-NFAT signaling and cardiac hypertrophic remodeling. Also, our findings support the hypothesis that mechanosensitivity of TRPC6 channels modulates cytosolic calcium transients and SR calcium load.

## Introduction

Transient receptor potential canonical (TRPC) 6 channels are non-selective cation channels expressed in the mammalian heart (Bon and Beech, 2013). The channels are permeable to Na^+^ and Ca^2+^, with six-fold higher permeability for Ca^2+^ than Na^+^ (Dietrich and Gudermann, 2014). TRPC6 channels can be activated by diacylglycerol (DAG) (Hofmann et al., 1999), a product of phospholipase C, and other DAG analogues (Aires et al., 2007). Other studies suggested that the level of TRPC6 at the plasma membrane increases upon muscarinic receptor stimulation and depletion of intracellular Ca^2+^ pool (Cayouette et al., 2004). TRPC6 channels are thought to be stretch-activated channels (SACs). SACs convert mechanical stimuli into electrical or chemical signals used in various mechanosensitive pathways (Martinac and Kloda, 2003; Sachs, 2010). SACs and mechanosensitive channels contribute to mechano-regulation of Ca^2+^ signaling in the normal and diseased heart (Friedrich et al., 2012). Stretch activation of TRPC6 channels was linked to increases in cytosolic Ca^2+^ concentration ([Ca^2+^]_i_) (Dyachenko et al., 2008). Also, TRPC6 was found to be a contributor to the adaptive stretch-induced slow force response, a slow increase in [Ca^2+^]_i_ and twitch force that develops during stretch (Yamaguchi et al., 2017). Furthermore, TRPC6 was suggested to be involved in cardiac systolic mechanosignaling (Seo et al., 2014). Despite these findings, the precise role of TRPC6 channels in Ca^2+^ signaling and its mechanical modulation in cardiomyocytes remains unclear. It is also unclear if in cardiomyocytes TRPC6 channels contribute to mechano-electrical feedback (MEF), i.e., alter cell electrophysiology in response to mechanical stimuli (Franz, 2000).

In cardiac disease, TRPC6 is a positive regulator of calcineurin-NFAT signaling and plays a critical role in angiotensin II-induced cardiac hypertrophy (Kuwahara et al., 2006; Onohara et al., 2006; Wu et al., 2010). TRPC6 was upregulated in response to activated calcineurin and pressure overload, further indicating a role in cardiac disease (Kuwahara et al., 2006). In pathological conditions, TRPC6 were suggested to underlie dysregulated [Ca^2+^]_i_ and force, as well as arrhythmogenicity (Seo et al., 2014). TRPC6 is thought to be a key contributor to the initiation of hypertrophy and heart failure (Yamaguchi et al., 2017). Endothelin-treated neonatal rat cardiomyocytes showed hypertrophy along with increased TRPC6 mRNA expression (Kiso et al., 2013). A study in mouse cardiomyocytes revealed that overexpression of TRPC6 causes spontaneous cardiac hypertrophy and remodeling (Xie et al., 2012).

The involvement of TRPC6 in cardiac diseases has made it a target for treatment. Deletion of TRPC6 prevented stress-induced remodeling in mice (Xie et al., 2012). The enzyme Klotho reduced TRPC6 currents in cardiomyocytes. Also, Klotho inhibited TRPC6 currents in HEK293 and L6 cell lines independently of fibroblast growth factors (Wright et al., 2019). Studies on human cardiac fibroblasts suggested that knockdown of TRPC6 reduces 1-oleoyl-2-acetyl-sn-glycerol-induced Ca^2+^ entry (Ikeda et al., 2013). Drug blockade of TRPC6 reversed the excessive slow force response in dystrophic myocardium (Seo et al., 2014).

Localization of TRPC6 within cardiomyocytes is dependent on developmental stages and species (Ahmad et al., 2017). A study in rat ventricular myocytes suggested that in neonates TRPC6 localizes in the cytoplasm and nuclear envelope, and in adult animals to z-lines, intercalated discs and the nucleus (Jiang et al., 2014). TRPC6 expression was highest during the fetal stage and decreased after birth, with similar levels in neonatal and adult animals. In contrast, studies on mouse ventricular myocytes suggested TRPC6 location in the t-system and plasma membrane (Dyachenko et al., 2008; Mohl et al., 2011). These differences stress the importance of understanding TRPC6 localization when interpreting functional measurements.

In this study, we aimed at understanding effects of upregulation of TRPC6 on Ca^2+^ signaling in cardiac myocytes. We studied neonatal rat ventricular myocytes (NRVMs) infected with adenoviral constructs to modulate TRPC6 expression. We applied confocal microscopy and super-resolution imaging to identify TRPC6 localization. We measured [Ca^2+^]_i_ transients and Ca^2+^ load of sarcoplasmic reticulum (SR) using Ca^2+^-sensitive dyes and spinning disk confocal microscopy. We applied computational modeling to qualitatively reproduce experimental findings and predict effects of upregulation or increased activity of TRPC6 on Ca^2+^ signaling.

## Methods

All studies were conducted at the University of Utah in accordance with National Institute of Health Guidelines for the Care and Use of Animals and reviewed by the Institutional Animal Care and Use Committee.

### Preparation, Adenoviral Infection, and Culture of NRVMs

Sprague Dawley rat dams were obtained from Charles River (Wilmington, MA, USA). Ventricular myocytes were enzymatically isolated from 1-day old rats (NCIS, Worthington Biochemical Corporation, Lakewood, NJ, USA). NRVMs were separated from fibroblasts by cell suspension to take advantage of fibroblast rapid adhesion and recovering myocyte suspension. Myocytes were counted and plated at 75,000 cells per 0.95 cm^2^ in a 48 well tissue culture plate containing No. 0 coverslips treated with fibronectin. NRVMs were infected 24 h after plating with an adenoviral vector containing human TRPC6 attached to enhanced green fluorescent protein (eGFP) fused to the C-terminal and 6X HIS tag fused to the N-terminal (TRPC6-eGFP) at 10 multiplication of infection (MOI). We applied also an eGFP vector (Cat No. 1060) at 20 MOI as control. Furthermore, we infected NRVMs with shRNA TRPC6 with eGFP tag (shRNA-TRPC6-eGFP, Cat No. shADV-226,546) at 100 MOI to silence native TRPC6 expression. The virus was removed 24 h after infection. Infected and washed cells were maintained and cultured for an additional 24–48 h in a humidity and CO_2_-controlled incubator at 37°C. All viral vectors were produced by Vector Biolabs (Malvern, PA, USA) with a backbone of type 5 (dE1/E3). All eGFP tags were under a CMV promoter.

### Western Blotting

Cultured cells were lysed in RIPA buffer. Protein concentration in each sample was determined using Pierce BCA protein assay kit (23227, ThermoFisher). RIPA buffer, 2-Mercaptoethanol (BME), and 6x gel loading dye were added to the protein lysate to create gel ready samples at 1 μg/μl. Samples were heated at 70°C for 10 min to reduce protein. Protein was loaded on an 8% Bis-Tris Plus Gel (NW00082, Thermo Fisher) and electrophoresed in MOPS running buffer (B001, Invitrogen) at 200 V for 35 min. Protein was transferred onto 0.45 μm nitrocellulose membrane in Tris-Glycine-Methanol buffer at 250 mA for 1 h. The transferred blot was blocked in 5% (w/v) non-fat milk in Tris-Buffered Saline with Tween 20 (TBS-T) for 60–90 min at room temperature. Blots were incubated with primary anti-TRPC6 (LS-C19628) or anti-eGFP (AB6556) antibodies in blocking solution @1:1000 dilution and left overnight at 4°C. After washing in TBS-T, goat anti-rabbit secondary antibody was applied at 1:50 k in TBS-T, while Precision Protein StrepTactin-HRP (1610380, BioRad) was applied at 1:100 k to stain for the molecular weight ladder. Blots were incubated in WesternBright ECL HRP substrate kit (K012045, Advansta, San Jose, CA, USA) for 2 min and imaged on Bio-Rad Image (Bio-Rad).

### Immunolabeling of NRVMs

Coverslip plated NRVMs were fixed with 1% paraformaldehyde (PFA) for 15 min at room temperature and then washed in phosphate-buffered saline (PBS) and stored at 4°C for immunolabeling. NRVMs were permeabilized with 0.3% Triton X-100 (VWR International, Radnor, PA, USA) for 18 min and bathed in image-iT Fx Signal Enhancer (I36933, Thermo Fisher) for 30 min. Cells were blocked in 10% normal donkey serum (D9663, Millipore, Billerica, MA, USA) for 60 min at room temperature. Primary antibody for TRPC6 (LS-C19628, LifeSpan BioSciences, Seattle, WA, USA) was incubated overnight in 2% normal donkey serum incubation solution at 4°C. The next day primary antibodies were triple-washed in PBS for 15 min. Secondary antibodies were applied for 60 min at room temperature. For confocal microscopy, the secondary antibody used was a donkey anti-rabbit conjugated to Alexa Fluor 647 (A31573, Thermo Fisher). Non-specific secondary labeling was controlled by similarly labeling NRVMs while omitting primary antibody. Cells were then incubated with DAPI (D3571, Thermo Fisher) for 15 min to stain the nuclei and rinsed in PBS to be held at 4°C for imaging. For super-resolution microscopy, an anti-GFP nanobody conjugated to Alexa Fluor 647 was applied (gb2AF647, Chromotek, Planegg-Martinsried, Germany).

### Confocal Microscopy, Image Acquisition, and Image Processing

Fixed and labeled NRVM coverslips were placed in PBS and imaged using a Leica SP8 confocal microscope (Leica Microsystems, Wetzlar, Germany). Two dimensional images were acquired using a GaAsP-HyD detector and a 40x oil immersion lens (numerical aperture 1.2) with a 0.1 × 0.1 μm pixel size. DAPI was excited with a 405 nm laser and emission collected at 410–550 nm. Fluorescence of eGFP was excited with a 488 nm laser and emission collected at 491–610 nm. Alexa Fluor 647 conjugated antibody was excited with a 633 nm laser and emission collected at 638–775 nm. All samples were imaged with identical imaging parameters. We used sequential framing to avoid simultaneous excitation of fluorophores and minimize cross-talk. Images were processed for noise reduction and background correction, then visualized with the same intensity ranges for comparison.

### Super-resolution Microscopy

Three-dimensional single-molecule localization microscopy was performed using a Vutara 352 (Bruker Corporation, Middleton, WI). Fixed and labeled TRPC6-eGFP NRVM coverslips were immersed in a blinking solution containing 20 mM MEA, 1% (v/v) 2-Mercaptoethanol, and an oxygen scavenging system (glucose oxidase and catalase) in a buffer of 50 mM Tris, 10 mM NaCl, and 10% (w/v) glucose. We applied a 640 nm excitation laser and 405 nm activation laser. A 60x water immersion lens (numerical aperture: 1.2) was used to collect 10,000 images with 20 ms exposure. We acquired image stacks with a size of approximately 18 μm × 18 μm × 2 μm. Localizations with less than median photon count and greater than median radial precision were filtered out.

### Measurement of [Ca^2+^]_i_ in NRVMs

[Ca^2+^]_i_ in infected NRVMs 48–72 h post-infection was measured using the ratiometric Ca^2+^ fluorescent dye indo-1 (I1203, Thermo Fisher). We applied an X-Light V2 spinning disk equipped with a Photometrics Prime 95B camera and an OptoSplit III LS (NCI Micro, Brooklyn Park, MN, USA). The spinning disk setup was built on a Zeiss confocal microscope (Carl Zeiss, Jena, Germany) equipped with a 40x oil lens and controlled with MetaMorph software (Molecular Devices, San Jose, CA, USA). We used a 365 ± 5 nm UV laser (ENTCII-653, Coherent, Santa Clara, CA, USA) for excitation of indo-1. Excitation and emission signals were split by a 387 nm long pass dichroic mirror (Chroma, Bellows Falls, VT, USA). Dual-wavelength emissions for the bound and unbound dye were split using a 440 nm dichroic mirror before band pass filtering (405 ± 15 and 485 ± 12.5 nm, respectively).

Cells were loaded with 20 μM indo-1 for 30 min at 37°C, then washed for 15–30 min before imaging. The cells were superfused with Tyrode solution (in mM: 126 NaCl, 4.4 KCl, 1 MgCl_2_, 24 HEPES, 11 D-Glucose, 12.5 NaOH, 1 CaCl_2_ and 0.7 probenecid) at room temperature (22 ± 1°C). We electrically paced the cells at 0.5 Hz until steady state was achieved. The image acquisition began with 10 s of 0.5 Hz pacing, followed by 10 s of 1 Hz pacing. We rapidly applied 20 mM caffeine to cause SR Ca^2+^ release after 10 s of 1 Hz pacing.

We obtained sequences of images at a rate of 50 Hz and with a pixel width and height of 0.278 μm. Images comprised dual-wavelength emissions for *F*_405,camera_ and *F*_485,camera_. The signals were registered for image processing and data extraction. Images from the camera *F*_405,camera_ and *F*_485,camera_ were corrected by subtraction of the camera background *F*_405,bg_ and *F*_485,bg_, respectively. We also corrected for cell autofluorescence and eGFP bleed-through for the two wavelength ranges, *F*_405,cell_ and *F*_485,cell_. We measured autofluorescence and eGFP bleed-through in NRVMs for all groups in the absence of indo-1. The corrected *F*_405_ and *F*_485_ images were calculated by:

$$F_{405}=F_{405,\mathrm{camera}}-F_{405,bg}-F_{405,\mathrm{cell}}$$

$$F_{485}=F_{485,\mathrm{camera}}-F_{485,bg}-F_{485,\mathrm{cell}}$$

Using our camera settings, *F*_405,*bg*_ and *F*_485,*bg*_ were 99.

Regions in the imaged cells were manually cropped to create a mask for calculation of *F*_405_ and *F*_485_ (Supplementary Figure S1). The ratio of signal from indo-1 with bound and unbound Ca^2+^ was determined:

$$F=\frac{F_{405}}{F_{485}}$$

Transients of [Ca^2+^]_i_ were calculated from F. We fit a first-order exponential function to measure the decay rate constant (*T_Decay_*) of [Ca^2+^]_i_ transients under each pacing rate. SR load was determined by measuring the amplitude of the caffeine-induced peak (Supplementary Figure S2). Cells with multiple or delayed caffeine peaks were excluded from the analysis.

### Calibration of [Ca^2+^]_i_ Measurements

NRVMs from the eGFP, TRPC6-eGFP, and shRNA-TRPC6-eGFP groups were loaded with indo-1 as described in Section “Measurement of [Ca^2+^]_i_ in Neonatal Rat Ventricular Myocytes.” Cells were bathed in a 0 mM Ca^2+^ solution containing (in mM): 126 NaCl, 4.4 KCL, 1 MgCl_2_, 11 D-Glucose, 24 HEPES, 12.9 NaOH, 10 EGTA, 0.7 probenecid, and 0.01 ionomycin (407,952, EDM MilliPore) for 15 min at 37°C and then rinsed in the same solution without ionomycin. Cells were imaged as described in Section “Measurement of [Ca^2+^]_i_ in Neonatal Rat Ventricular Myocytes,” while in 0 mM Ca^2+^ solution containing 5 μM ionomycin and 40 mM 2,3-butanedione 2-monoxime (BDM) for 2–3 min before rapidly switching to a Tyrode solution containing 2 mM Ca^2+^ and 40 mM BDM. *F* at 0 and 2 mM Ca^2+^ determined *F*_min_ and *F*_max_, respectively, in the calibration equation (Grynkiewicz et al., 1985):

$${\left[{Ca}^{2+}\right]}_{i}=K_{d}\frac{F-F_{\text{min}}}{F_{\text{max}}-F}\;\frac{S_{f2}}{S_{b2}}$$

Here, *K*_d_ is the equilibrium dissociation constant for Ca^2+^, set to 250 nM based on literature (Grynkiewicz et al., 1985; Ikenouchi et al., 1991). *S*_f2_ and *S*_b2_ represent *F*_485_ during 0 and 2 mM Ca^2+^, respectively. Traces for calibration were extracted with the same methods as for [Ca^2+^]_i_ measurements in paced cells.

### Modeling of Sarcolemmal Ca^2+^ Leak in NRVMs

We used a mathematical model of NRVM electrophysiology for qualitative comparison to our experimental results (Korhonen et al., 2009). Current through TRPC6 channels (*I*_TRPC6_) was modeled as an additional sarcolemmal leak Ca^2+^ current:

$$I_{\mathrm{TRPC}6}=G_{\mathrm{TRPC}6}\left(V_{m}-E_{Ca}\right)$$

where *G*_TRPC6_ is the Ca^2+^ conductance for TRPC6 channels in the sarcolemma, *V*_m_ is the membrane voltage and *E*_Ca_ is the Ca^2+^ Nernst voltage. *E*_Ca_ was determined by:

$$E_{Ca}=\frac{RT}{2F}\ln\frac{{\left[{Ca}^{2+}\right]}_{o}}{{\left[{Ca}^{2+}\right]}_{\mathrm{subSL}}}$$

with the gas constant *R*, temperature *T*, Faraday constant *F*, extracellular Ca^2+^ concentration [Ca^2+^]_o_ and subsarcolemmal Ca^2+^ concentration [Ca^2+^]_subSL_.

In our simulations with the revised model, we varied *G*_TRPC6_ to simulate increased TRPC6 expression. For pacing, i.e., triggering of action potentials, we applied intracellular current of −80 pA/pF for a duration of 0.5 ms at 0.5 Hz or 1 Hz for 1 min. [Ca^2+^]_i_ transients from the final beat were analyzed. *T_Decay_* was calculated by fitting an exponential function to the decay phase of the transient. Application of caffeine was simulated 5 s following cessation of 1 Hz pacing. The effect of caffeine was modeled as previously described (Korhonen et al., 2009) by setting RyR Ca^2+^ flux to a large constant value and SERCA Ca^2+^ uptake to 0 μM/ms.

We also used the model to explore effects beyond those measured in our experiments. Ca^2+^ fluxes through other ion channels, exchanges, and pumps were calculated to evaluate how their contributions change of Ca^2+^ dynamics in response to changes in *G*_TRPC6_. Currents (*I*_channel_) through sarcolemmal Ca^2+^ channels and exchangers were converted to Ca^2+^ fluxes (*J*_channel_):

$$J_{\mathrm{channel}}=I_{\mathrm{channel}}\frac{A_{\mathrm{cap}}C_{m}}{2F\times 10^{-6}\mathrm{\mu l}/\mathrm{pl}\;}$$

where *A*_cap_ is the capacitive membrane area (1.38544 × 10^−5^ cm^2^), *C*_m_ is the specific membrane capacitance (1 μF/cm^2^), and *F* is Faradays constant of 96.5 C/mmol. The units of the resultant fluxes are μM/s, where the concentration is defined per cytosol volume. Fluxes through RyR and SERCA channels were converted to account for this definition. We integrated each flux over 2 and 1 s for 0.5 and 1 Hz pacing, respectively, to determine its effect on [Ca^2+^]_i_.

### Statistical Analyses

Data are presented as mean ± standard error. Data analysis was performed in MATLAB version R2019a or higher (Mathworks Inc., Natick, MA, USA). Differences in experimental data were assessed using the *t*-test and considered significant for *p* less than 0.05.

## Results

### Validation of Adenoviral Infection and Antibodies

**Figure 1:**
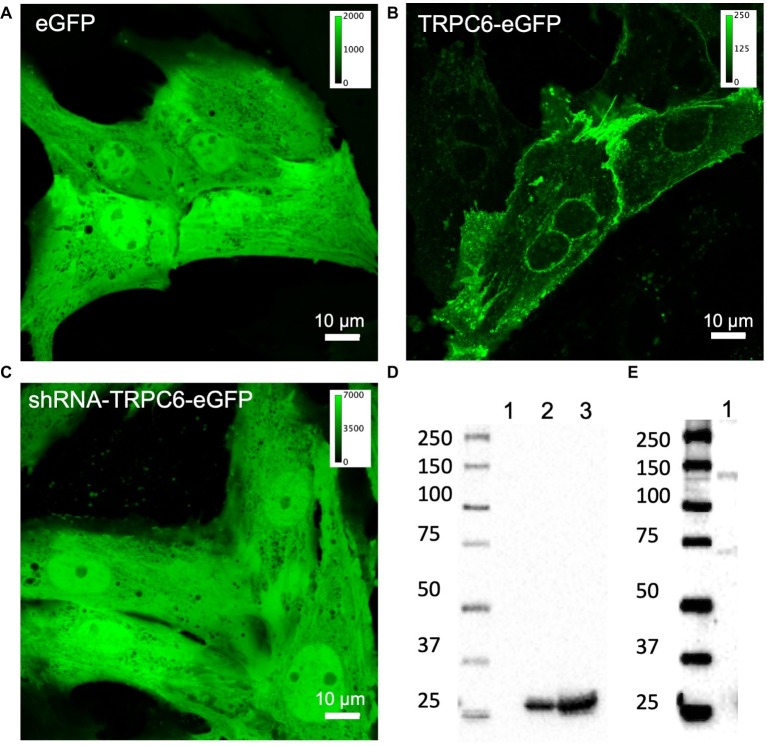
Confocal microscopic images of living NRVMs infected with **(A)** eGFP at 20 MOI, **(B)** TRPC6-eGFP at 10 MOI, and **(C)** shRNA-TRPC6-eGFP at 100 MOI. eGFP and shRNA-TRPC6-eGFP infected cells exhibit a diffuse cytosolic signal. In TRPC6-eGFP cells, signal is localized to sarcolemma and nuclear membrane. Colormap for each signal is added for comparison of fluorescence intensities between groups. **(D)** Western blotting with anti-eGFP antibody (AB6556) of NRVMs expressing TRPC6-eGFP (lane 1), eGFP (lane 2), and shRNA-TRPC6-eGFP (lane 3). Expression of eGFP at ~30 kDa band was detected in eGFP and shRNA-TRPC6-eGFP cells. **(E)** Longer exposure of lane 1 confirms TRPC6-eGFP expression at the expected 135 kDa molecular weight of the construct.

We confirmed adenoviral infection and expression of eGFP, TRPC6-eGFP, and shRNA-TRPC6-eGFP constructs in live NRVMs using confocal microscopy (Figures 1A–C). Expression of TRPC6-eGFP concentrated along the sarcolemma and was also visible near the nucleus (Figure 1B, *n*_images_ = 10, *n*_cells_ = 25). Fluorescence in eGFP (*n*_images_ = 5, *n*_cells_ = 20) and shRNA-TRPC6-eGFP (*n*_images_ = 3, *n*_cells_ = 10) infected cells was present in the entire myocyte (Figures 1A,C). Western blotting with anti-eGFP antibody showed bands at ~30 kDa for eGFP and shRNA-TRPC6-eGFP infected cells (Figure 1D). Western blots of TRPC6-eGFP cells showed a band at ~135 kDa (Figure 1E), which was not present for eGFP or shRNA-TRPC6-eGFP cells (Figure 1D), even at higher exposure (Supplementary Figure S3). Probing NRVMs with anti-TRPC6 antibody LS-C19628 showed bands at ~135 kDa only for the TRPC-eGFP group (Supplementary Figure S4, *n*_litters_ = 3), similar to the anti-eGFP western blot (Figure 1E).

### Localization of TRPC6 in NRVMs

**Figure 2:**
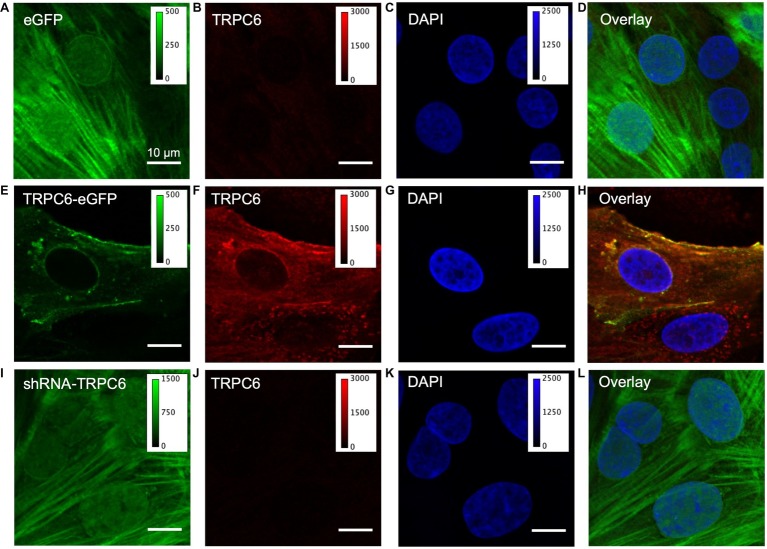
Confocal microscopic images of fixed infected NRVMs. **(A)** NRVM infected with eGFP at 20 MOI present an intracellular eGFP distribution. **(B)** TRPC6 labeling with LS-C19628 antibody shows marginal signal. **(C)** DAPI used as a marker for the nucleus. **(D)** Overlay of **(A–C)**. **(E)** NRVMs infected with TRPC6-eGFP at 10 MOI present signal at the sarcolemma. **(F)** TRPC6 labeling shows high fluorescence signal at the sarcolemma and nuclear membrane. **(G)** DAPI as marker for the nucleus. **(H)** Overlay of **(E–G)** shows high colocalization of TRPC6-eGFP and anti-TRPC6 antibody labeling, suggesting successful detection of our vector by the antibody. **(I)** NRVM infected with shRNA-TRPC6-eGFP at 100 MOI present diffuse, very bright intracellular eGFP signal. **(J)** TRPC6 labeling shows marginal fluorescence. **(K)** DAPI as a marker for the nucleus. **(L)** Overlay of **(I–K)**. Imaging of eGFP, TRPC6, and DAPI in the three experimental groups was done with identical settings. Colormap for each signal is added for comparison of fluorescence intensities between groups. TRPC6 labeling had much higher fluorescence in TRPC6-eGFP than eGFP and shRNA-TRPC6-eGFP cells. Scale bar in **(A)** applies to **(B–L)**.

We next studied fixed NRVMs from the experimental groups labeled with anti-TRPC6 antibody and DAPI. In the eGFP group (*n*_cells_ = 40, *n*_images_ = 11, *n*_litters_ = 5), eGFP signal exhibited a diffuse, in part sarcomeric pattern, and also localized to the nuclear membrane and nucleus (Figure 2A). Native TRC6 labeling with LS-C19628 yielded marginal signal (Figure 2B). DAPI labeling marked the nucleus of myocytes (Figure 2C). An overlay of the images from NRVMs expressing eGFP is shown in Figure 2D. In NRVM expressing TRPC6-eGFP (*n*_cells_ = 40, *n*_images_ = 18, *n*_litters_ = 5), the TRPC6-eGFP signal was associated with the sarcolemma and nuclear membrane as well as near the nucleus (Figure 2E). Anti-TRPC6 antibody signal was in the sarcolemma and irregularly distributed throughout the cell (Figure 2F). DAPI labeled the nuclei (Figure 2G). Overlay of TRPC6-eGFP and anti-TRPC6 images showed strong overlap of fluorescence in yellow (Figure 2H). In NRVMs expressing shRNA-TRPC6-eGFP (*n*_cells_ = 50, *n*_images_ = 5, *n*_litters_ = 2), eGFP signal was similar to signal in eGFP infected cells (Figure 2I). Labeling with anti-TRPC6 antibody yielded marginal signals (Figure 2J). DAPI marked the nuclei (Figure 2K). Overlay images were similar as in the eGFP group (Figure 2L). Brightness-adjusted images corresponding to Figures 2B,F,J are presented in Supplementary Figures S5A–C, respectively. Quantification of anti-TRPC6 antibody signal in these images is consistent with the visual impression that TRPC6-eGFP infection leads to a strong increase vs. eGFP infection (Supplementary Figure S5D). Marginal anti-TRPC6 antibody signal from eGFP infected NRVMs was further reduced in shRNA-TRPC6-eGFP infected NRVMs. Secondary antibody-only control showed negligible labeling in eGFP and TRPC6-eGFP NRVMs (Supplementary Figure S6).

**Figure 3:**
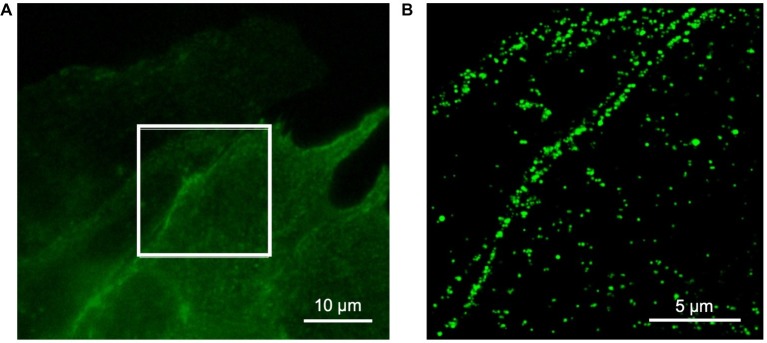
Super-resolution microscopy of fixed NRVM infected with TRPC6-eGFP at 10 MOI and labeled with anti-eGFP nanobody. **(A)** Wide-field reference image of eGFP signal. **(B)** Super-resolution image of TRPC6-eGFP signal from box in **(A)**. The TRPC6-eGFP pattern suggests sarcolemmal localization.

We performed super-resolution microscopy using an anti-eGFP nanobody to further assess location of TRPC6-eGFP. An example image is shown in Figure 3. The images supported sarcolemmal localization of the TRPC6-eGFP construct.

### Calibration of Indo-1

We assessed 30 regions in four images from eGFP, TRPC6-eGFP, and shRNA-TRPC6-eGFP infected cells without dye under indo-1 settings. *F*_405,cell_ signal ranged from 3.3 to 3.7 ± 0.2 for all groups. *F*_485,cell_ was 12.2 ± 0.2, 12.5 ± 0.2, and 20.8 ± 0.5 for eGFP, TRPC6-eGFP, and shRNA-TRPC6-eGFP cells.

**Figure 4:**
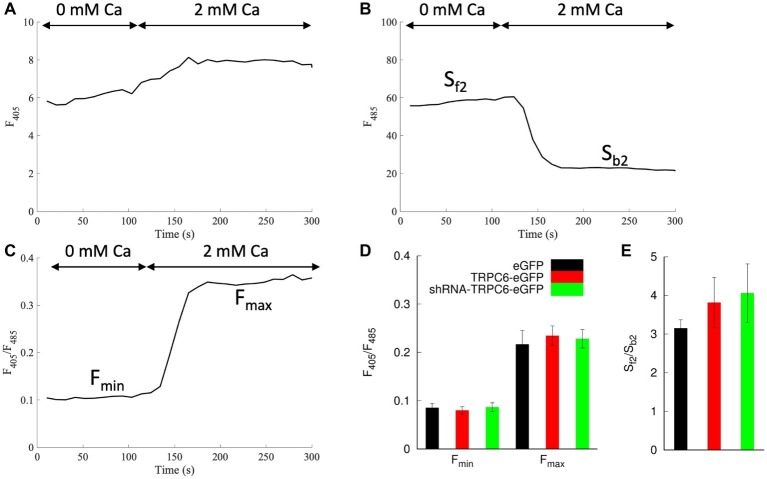
Indo-1 calibration protocol in NRVMs bathed in 10 μM ionomycin. Example trace shows **(A)** *F*_405_, **(B)** *F*_485_, and **(C)** *F*_405_/*F*_485_ during application of a bath solution with 0 and 2 mM Ca^2+^. **(D)** *F*_min_ and *F*_max_ were calculated from *F*_405_/*F*_485_ for 0 and 2 mM Ca^2+^ application, respectively. *F*_min_ and *F*_max_ for all groups were not significantly different. **(E)** Average *S*_f2_/*S*_b2_ was calculated as the ratio from the *F*_485_ emission during the 0 Ca^2+^ and 2 mM Ca^2+^. *S*_f2_/*S*_b2_ in all groups was not significantly different.

We applied infected NRVMs loaded with indo-1 for calibration of [Ca^2+^]_i_ measurements. We applied ionomycin to measure indo-1 signals in NRVMs bathed in low and high calcium (Figure 4). Example traces of *F*_405_, *F*_485_, and *F*_405_/*F*_485_ are presented in Figures 4A–C, respectively. *F*_min_ for eGFP, TRPC6-eGFP, and shRNA-TRPC6-eGFP was 0.085 ± 0.009 (*n*_cells_ = 7), 0.08 ± 0.008 (*n*_cells_ = 8), and 0.087 ± 0.009 (*n*_cells_ = 5), respectively (Figure 4D). *F*_max_ for eGFP, TRPC6-eGFP, and shRNA-TRPC6-eGFP was 0.22 ± 0.03, 0.24 ± 0.02, and 0.23 ± 0.02, respectively (Figure 4D). *S*_f2_/*S*_b2_ measured from the *F*_485_ signal was 3.2 ± 0.2, 3.8 ± 0.7, and 4.1 ± 0.8 for the eGFP, TRPC6-eGFP, and shRNA-TRPC6-eGFP, respectively (Figure 4E). Since we did not find significant differences in any of the measurements between the three groups, we averaged measures of cells from all the groups and calculated *F*_min_, *F*_max_, and *S*_f2_/*S*_b2_ to 0.084, 0.227, and 3.645, respectively.

### Measurements of [Ca^2+^]_i_ in NRVMs

We measured *F*_405_/*F*_485_ in NRVMs expressing eGFP (*n*_cells_ = 19, *n*_litters_ = 6), TRPC6-eGFP (*n*_cells_ = 19, *n*_litters_ = 5), and shRNA-TRPC6-eGFP (*n*_cells_ = 16, *n*_litters_ = 4). We present examples of *F*_405_/*F*_485_ transients from NRVMs expressing eGFP and TRPC6-eGFP under 0.5 Hz (Supplementary Figure S7A) and 1 Hz (Supplementary Figure S7B). Diastolic *F*_405_/*F*_485_ increased from 0.097 ± 0.004 in eGFP to 0.113 ± 0.005 in TRPC6-eGFP (*p* = 0.03) at 0.5 Hz, and from 0.105 ± 0.004 to 0.12 ± 0.005 at 1 Hz (*p* = 0.02) (Supplementary Figure S7C). Systolic *F*_405_/*F*_485_ of TRPC6-eGFP cells increased to 0.144 ± 0.005 compared to eGFP 0.124 ± 0.005 (*p* = 0.004) at both 0.5 Hz and 1 Hz (Supplementary Figure S7D). The amplitude of [Ca^2+^]_i_ transient was not significantly different between eGFP and TRPC6-eGFP cells paced at 0.5 Hz (0.026 ± 0.003 vs. 0.031 ± 0.005, *p* = 0.36) or 1 Hz (0.019 ± 0.003 vs. 0.024 ± 0.004, *p* = 0.35). Also, SR load was not different before calibration (Supplementary Figure S7E).

**Figure 5:**
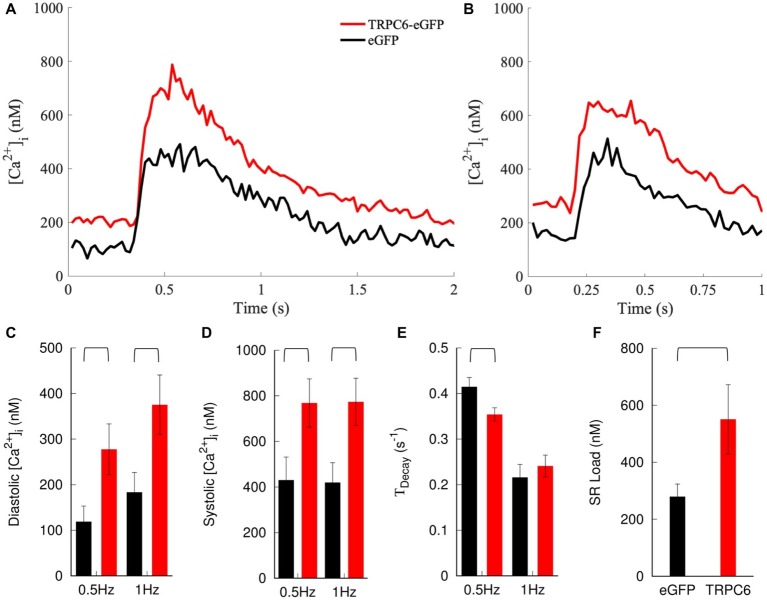
Measurement and analysis of [Ca^2+^]_i_. Example traces for NRVMs expressing eGFP or TRPC6-eGFP and paced at **(A)** 0.5 and **(B)** 1 Hz. Expression of TRPC6-eGFP caused a positive shift in [Ca^2+^]_i_ transients vs. eGFP at both pacing rates. **(C)** Diastolic and **(D)** systolic [Ca^2+^]_i_ increased in TRPC6-eGFP vs. eGFP cells for both pacing rates. **(E)** *T_Decay_* decreased in TRPC6-eGFP vs. eGFP cells for 0.5 Hz pacing. **(F)** SR load increased in NRVMs expressing TRPC6-eGFP vs. eGFP.

We applied the calibration to the measured *F*_405_/*F*_485._ NRVMs expressing TRPC6-eGFP cells exhibited a positive shift in cytosolic [Ca^2+^]_i_ transients compared to eGFP cells at both 0.5 and 1 Hz pacing rates (Figures 5A,B). Diastolic [Ca^2+^]_i_ levels increased in TRPC6-eGFP compared to eGFP cells at 0.5 Hz (278 ± 56 vs. 119 ± 34 nM, *p* = 0.02) and 1 Hz (375 ± 65 vs. 184 ± 43 nM, *p* = 0.02) (Figure 5C). Systolic [Ca^2+^]_i_ also increased in TRPC6-eGFP vs. eGFP cells at both 0.5 Hz (769 ± 106 vs. 430 ± 101 nM vs., *p* = 0.03) and 1 Hz (774 ± 103 vs. 420 ± 87 nM, *p* = 0.01) (Figure 5D). The amplitude of [Ca^2+^]_i_ transients was not significantly different between eGFP and TRPC6-eGFP at 0.5 or 1 Hz (data not shown). *T_Decay_* was larger for eGFP than TRPC6-eGFP cells at 0.5 Hz (0.42 ± 0.02 vs. 0.35 ± 0.02 s^−1^, *p* = 0.02), but not at 1 Hz (0.22 ± 0.03 vs. 0.24 ± 0.02 s^−1^, *p* = 0.5) (Figure 5E). Furthermore, the SR Ca^2+^ load increased from 279 ± 44 nM in eGFP to 551 ± 121 nM in TRPC6-eGFP (*n*_cells_ = 11, *n*_litters_ = 4–5, *p* = 0.048) (Figure 5F).

NRVMs infected with shRNA-TRPC6-eGFP showed similar features as eGFP infected cells (Supplementary Figure S8) and differences were not significant. Diastolic F_405_/F_485_ in shRNA-TRPC6 was 0.09 ± 0.004 (*p* = 0.3) and 0.104 ± 0.005 (*p* = 0.89) for 0.5 and 1 Hz, respectively (Supplementary Figure S8A). Systolic *F*_405_/*F*_485_ was 0.125 ± 0.006 (*p* = 0.82) and 0.127 ± 0.006 (*p* = 0.66) at 0.5 and 1 Hz, respectively (Supplementary Figure S8B). Calibrated diastolic [Ca^2+^]_i_ for shRNA-TRPC6-eGFP was 76.5 ± 31.8 (*p* = 0.37) and 176.6 ± 48.1 (*p* = 0.92) for 0.5 and 1 Hz pacing, respectively (Supplementary Figure S8C). Systolic [Ca^2+^]_i_ was 447.9 ± 85.2 (*p* = 0.9) and 502.3 ± 107.8 (*p* = 0.55) for 0.5 and 1 Hz pacing, respectively (Supplementary Figure S8D). *T_Decay_* was not significantly different at 0.5 Hz (0.43 ± 0.04, *p* = 0.7) or 1 Hz (0.2 ± 0.02, *p* = 0.6) vs. eGFP.

### Modeling of Sarcolemmal Ca^2+^ Leak in NRVMs

**Figure 6:**
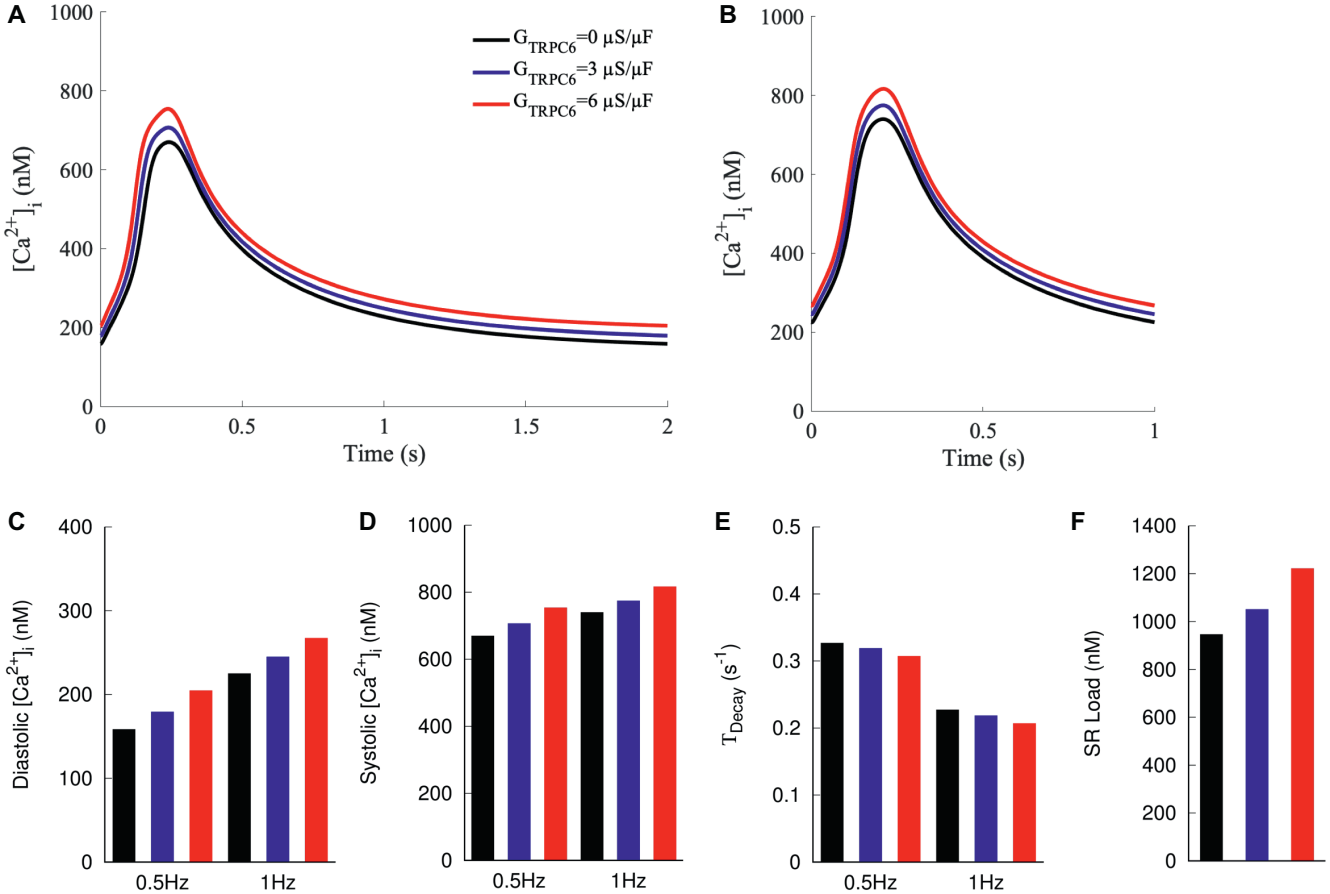
Computer model of NRVM with currents through TRPC6 channels modeled as sarcolemmal leak with G_TRPC6_ at 0, 3, and 6 μS/μF. [Ca^2+^]_i_ at **(A)** 0.5 and **(B)** 1 Hz pacing. Increased G_TRPC6_ caused a positive shift in [Ca^2+^]_i_ transients at both 0.5 and 1 Hz pacing. **(C)** Diastolic and **(D)** systolic [Ca^2+^]_i_ at 0.5 and 1 Hz increased for increased G_TRPC6_. **(E)** *T_Decay_* decreased with increased G_TRPC6_. **(F)** SR load increased with increasing G_TRPC6_.

We applied a mathematical model of NRVM electrophysiology to qualitatively reproduce findings of our experimental investigations. Increased TRPC6 expression was simulated by increasing G_TRPC6_ from 0 to 3 or 6 μS/μF. Increased *G*_TRPC6_ raised [Ca^2+^]_i_ during both diastole and systole (Figures 6A–D). For 0.5 Hz pacing, diastolic [Ca^2+^]_i_ was 159, 180, and 205 nM for *G*_TRPC6_ of 0, 3, and 6 μS/μF, respectively. Diastolic [Ca^2+^]_i_ increased for pacing with 1 Hz to 225, 245, and 267 nM for *G*_TRPC6_ of 0, 3, and 6 μS/μF, respectively (Figure 6C). Systolic [Ca^2+^]_i_ was 670, 707, and 754 nM for 0.5 Hz pacing and 740, 775, and 817 nM for 1 Hz pacing (Figure 6D) with *G*_TRPC6_ of 0, 3, and 6 μS/μF, respectively. The amplitude of [Ca^2+^]_i_ was 512, 528, and 550 nM for 0.5 Hz pacing, and 516, 530, and 550 nM for 1 Hz pacing. For both pacing rates, *T_Decay_* decreased with increasing *G*_TRPC6_ (Figure 6E). *T_Decay_* for 0.5 Hz pacing was 0.327, 0.319, and 0.307 s^−1^ for *G*_TRPC6_ of 0, 3, and 6 μS/μF, respectively. *T_Decay_* for 1 Hz pacing was 0.227, 0.219, and 0.207 s^−1^ for *G*_TRPC6_ of 0, 3, and 6 μS/μF, respectively. The amplitude of [Ca^2+^]_i_ following simulation of caffeine application after 1 Hz pacing increased with increased *G*_TRPC_, with 947, 1,052, and 1,222 nM for *G*_TRPC6_ of 0, 3, and 6 μS/μF, respectively (Figure 6F).

We used the model to predict effects of increased TRPC6 activity not explored in our experiments (Supplementary Figures S9, S10). Increased *G*_TRPC6_ resulted in an elevated resting *V*_m_ (Supplementary Figure S9A). For 0.5 Hz pacing, resting *V*_m_ was −69.95, −68.05, and − 66.03 mV for *G*_TRPC6_ of 0, 3, and 6 μS/μF, respectively. *V*_m_ for 1 Hz was −69.45, −67.91, and − 66.28 mV for G_TRPC6_ of 0, 3, and 6 μS/μF, respectively (Supplementary Figure S9E). Other major ion currents were assessed by calculating the integrated Ca^2+^ flux during a single beat. Integrated flux of Ca^2+^ through TRPC6 during 0.5 Hz was 7.4 and 14.6 μM for *G*_TRPC6_ of 3 and 6 μS/μF, respectively. Integrated Ca^2+^ through TRPC6 during 1 Hz was 3.4 and 6.8 μM (Supplementary Figures S9B,F). Integrated Ca^2+^ flux through L-type calcium channels (LCCs) was 30.2, 28.6, and 27.4 μM at 0.5 Hz and 24.4, 23.5, and 22.6 μM at 1 Hz (Supplementary Figures S9C,G). For 0.5 Hz pacing, integrated Ca^2+^ flux through NCX was 54.3, 60.5, and 66.9 μM for *G*_TRPC6_ of 0, 3, and 6 μS/μF, respectively. For 1 Hz pacing, the integrated flux of Ca^2+^ out of the cell through NCX was 35.6, 38.2, and 40.9 μM for *G*_TRPC6_ of 0, 3, and 6 μS/μF, respectively (Supplementary Figures S9D,H). The integrated Ca^2+^ release into the cytosol from the SR through RyRs was 8.5, 9.7, and 11.2 μM at 0.5 Hz and 7.5, 8.1, and 8.8 μM at 1 Hz, for *G*_TRPC6_ of 0, 3, and 6 μS/μF, respectively (Supplementary Figures S10A,C). The integrated Ca^2+^ pumped back into the SR through SERCA was 8.7, 9.9, and 11.4 μM at 0.5 Hz and 7.6, 8.2, and 8.9 μM at 1 Hz for G_TRPC6_ of 0, 3, and 6 μS/μF, respectively (Supplementary Figures S10B,D).

## Discussion

Our study supports a role of TRPC6 in sarcolemmal Ca^2+^ signaling in cardiomyocytes. Cells infected with eGFP vector served as control, while TRPC6-eGFP infected cells overexpressed TRPC6. Our primary findings are that TRPC6-eGFP was associated with the sarcolemma and, in paced myocytes, TRPC6 expression increased [Ca^2+^]_i_ and SR Ca^2+^ load in a pacing rate-dependent manner. We explored shRNA-TRPC6-eGFP constructs to reduce TRPC6 expression, but our structural and functional studies suggested that TRPC6 is only marginally expressed in NRVMs.

### TRPC6-eGFP Localization

Our confocal and super-resolution imaging suggested that localization of TRPC6-eGFP is predominantly at the sarcolemma (Figures 1B, 2E, 3). We also found TRPC6-eGFP near the nuclear envelope, likely in the endoplasmic reticulum or Golgi apparatus, suggesting that the protein is on track for translocation and sarcolemmal integration. The eGFP and shRNA-TRPC6-eGFP constructs produced eGFP signals diffuse in the cytosol, indicating successful infection with the adenoviral vector (Figures 1A,C).

Western blotting with anti-eGFP antibody showed differences in the molecular weight detected in our experimental groups (Figures 1D,E). The TRPC6-eGFP group presented a band at ~135 kDa, which is explained by the TRPC6 molecular weight of ~105 kDa plus the weight of the conjugated eGFP of ~30 kDa. A 135 kDa band was not detected for eGFP or shRNA-TRPC6-eGFP, even at higher exposure (Supplementary Figure S3). The eGFP control and shRNA-TRPC6-eGFP groups only showed a band at 32 kDa for eGFP proteins. Differences of intensity of the bands between the groups are related to the difference in MOI for TRPC6-eGFP (10), eGFP (20), and shRNA-TRPC6-eGFP (100).

Fixation and immunolabeling led to changes in the eGFP patterns of eGFP and shRNA-TRPC6-eGFP infected NRVMs (Figures 2A,I), which are likely due to permeabilization of the sarcolemma and loss of eGFP from the cytosol, only leaving eGFP immobilized due to fixation. Similarities of TRPC6-eGFP patterns in fixed (Figure 2E) and living (Figure 1B) NRVMs further supported localization of the construct to the sarcolemma. Native TRPC6 signal in NRVMs infected with eGFP (Figure 2B) and shRNA-TRPC6-eGFP (Figure 2J) was marginal when compared under the same image settings used for TRPC6-eGFP NRVMs (Figure 2F) and in the brightness-adjusted images (Supplementary Figure S5). Secondary antibody only imaging presented even smaller signals (Supplementary Figure S6). The comparison indicates a pronounced expression of TRPC6-eGFP in the NRVMs in the context of marginal native TRPC6 expression.

### Anti-TRPC6 Antibody Labeling in Infected NRVMs

The applied anti-TRPC6 antibody (LS-C19628) was developed from a 14 amino acid synthetic peptide from amino acids 50–100 of the human TRPC6 sequence. The antibody was validated by the manufacturer for western blot and immunofluorescence in human samples as well as immunochemistry in mouse samples. Comparison of the TRPC6 protein sequence from human and rat (Genbank Accession No. NP_004612 and NP_446011, respectively) shows 93.88% homology. Weak homology was in particular within the first 60 and last 100 amino acids. Comparison of the protein sequence from mouse and rat (Genbank Accession No. NP_03886 and NP_446011, respectively) exhibited 98.17% homology, with amino acids 50–100 being conserved in both species.

Successful detection of our TRPC1-eGFP construct in western blots supports sensitivity of the antibody for human TRPC6 (Supplementary Figure S4). However, the antibody did not show a band at the expected molecular weight of 106 kDa for the native rat TRPC6 (Supplementary Figure S4). In our immunofluorescence images, the antibody presented high colocalization with eGFP in TRPC6-eGFP infected cells (Figure 2H). Also, we noticed a marginal immunofluorescence signal in eGFP infected cells (Supplementary Figure S5A) that was reduced in shRNA-TRPC6-eGFP NRVMs (Supplementary Figures S5C,D). These findings indicate that the antibody is not sensitive for rat TRPC6 in western blotting but detects rat TRPC6 in both, human and rat in immunofluorescence (Supplementary Figure S5D). We evaluated further anti-TRPC6 antibodies for western blotting and immunofluorescence (Supplementary Table S1). Supplementary Figure S11 presents a western blot using an antibody (AB-105845) that is capable of detecting rat TRPC6 and TRPC6-eGFP. However, this and another tested antibody (LS-B611) that targeted the region close to c-terminus were not capable of detecting TRPC6-eGFP in immunofluorescence. Our TRPC6-eGFP construct is based on fusion of eGFP to the TRPC6 c-terminus, which might interfere with antibody binding.

### [Ca^2+^]_i_ Calibration of Indo-1 Signals in the Presence of eGFP

Quantification of [Ca^2+^]_i_ from indo-1 signals required correction for camera background, autofluorescence, and eGFP bleed-through. Contribution of eGFP to *F*_485,cell_, but not *F*_405,cell_ signal, varied across the NRVMs depending on MOI. The difference in *F*_485,cell_ across our experimental groups strongly affected calculation of F. TRPC6-eGFP and eGFP infection at 10 and 20 MOI yielded similar levels of bleed-through as we found in uninfected NRVMs (data not shown). In contrast, shRNA-TRPC6-eGFP infection at 100 MOI almost doubled the bleed-through, suggesting that a high eGFP expression causes a fluorescence offset beyond the offset primarily explained by autofluorescence. We suggest that our approach for correction is useful in other studies with eGFP markers at high MOIs, where changes in the *F*_485_ signal must be accounted for.

Calibration using ionomycin and varying extracellular Ca^2+^ allowed us to calculate [Ca^2+^]_i_ accounting for the non-linear ratiometric fluorescence properties of indo-1. After corrections, eGFP, TRPC6-eGFP, and shRNA-TRPC6-eGFP infected cells yielded similar *F*_min_, *F*_max_, and *S*_f2_/*S*_b2_ (Figure 4). This indicates that our corrections were successful. Averaging measures from all cells gave a uniform parameter set for calculating [Ca^2+^]_i_.

### Comparison of [Ca^2+^]_i_ Measurements With Prior Work

In our eGFP NRVMs, diastolic [Ca^2+^]_i_ of 119 ± 34 nM at 0.5 Hz and 183 ± 43 nM at 1 Hz are close to previously measured diastolic [Ca^2+^]_i_ of 140 ± 11 nM for spontaneous beating 2- to 7-day-old NRVMs (Gomez et al., 1994). In these cells, systolic [Ca^2+^]_i_ ranged from 323 to 480 nM, comparable to the measurement of 430 ± 100 nM in our 4- to 5-day-old NRVMs. Other studies on paced rat cardiomyocytes reported systolic [Ca^2+^]_i_ in the range from 319 ± 37 to 454 ± 70 nM in 18-day fetal and rise to 743 ± 64 nM in adult rat (Seki et al., 2003). Previously reported *T_Decay_* at 0.5 Hz pacing ranged from 0.47 ± 0.1 s^−1^ in the 18-day fetus to 0.2 ± 0.02 s^−1^ in the adult rat, similar to our measured *T_Decay_* of 0.42 ± 0.02 s^−1^ at 0.5 Hz for eGFP NRVMs. Application of caffeine in our eGFP myocytes caused a 279 ± 44 nM rise in cytosolic Ca^2+^, which is within the range of example responses for 2- and 7-day-old NRVMs (Gomez et al., 1994).

### Increased TRPC6 Expression Leads to Increased [Ca^2+^]_i_ and SR Load in Paced Cells

Raw and calibrated measurements indicated an increase in [Ca^2+^]_i_ of TRPC6-eGFP compared to eGFP cells (Figure 5, Supplementary Figure S7). Diastolic and systolic [Ca^2+^]_i_ increased, but the amplitude did not, indicating only a offset in the overall transient. We qualitatively compared experimental findings with results from mathematical modeling assuming that TRPC6 causes sarcolemmal Ca^2+^ leak. In the model, diastolic and systolic [Ca^2+^]_i_ increased with increasing *G*_TRPC6_ (Figures 6A,B). The amplitude of [Ca^2+^]_i_ also increased, but to a small degree, which could explain why our measurements were not significantly different in the experimental groups.

We observed a pacing rate-dependent increase in diastolic [Ca^2+^]_i_ of NRVMs, which is in agreement with previous findings in adult rat (Dibb et al., 2007; Gattoni et al., 2016). In the model, 1 Hz pacing caused a 30, 36, and 42% increase in diastolic [Ca^2+^]_i_ vs. 0.5 Hz pacing for G_TRPC6_ of 0, 3, and 6 μS/μF, respectively (Figure 6C). Systolic [Ca^2+^]_i_ was also higher with 1 Hz pacing (Figure 6D), but interestingly, the amplitude of [Ca^2+^]_i_ transient remained mostly unchanged for both pacing rates for all *G*_TRPC6_ settings. Additionally, *T_Decay_* reduced with increased TRPC6 in both, experiments (Figure 5E) and simulations (Figure 6E). Also, *T_Decay_* was reduced for increased pacing rate.

Caffeine reduces the Ca^2+^ threshold for activation of RyR clusters and triggers release of the SR Ca^2+^ into the cytosol. The increase of [Ca^2+^]_i_ is often used as a measure of SR Ca^2+^ load. SR load was increased in TRPC6-eGFP vs. eGFP NRVMs (Figure 5F). Simulation of caffeine application also resulted in increased for increased *G*_TRPC6_ (Figure 6F).

Overall, the simulation results aligned with experimental findings, supporting the description of TRPC6 channels as sarcolemmal Ca^2+^ leak. The simulations also helped in mechanistic explanation of the experimental findings and predicted effects of TRPC6 activity not measured in our experiments. With the model, we evaluated Ca^2+^ flux through ion channels, pumps and exchangers. Ca^2+^ flux through TRPC6 channels was small (Supplementary Figures S9B,F) vs. LCC and NCX fluxes (Supplementary Figures 9C,D,G,H) but associated with notable changes of all Ca^2+^ fluxes. As a result of increased *G*_TRPC6_, LCC fluxes decreased (Supplementary Figures S9C,G). The integral efflux of Ca^2+^ through NCX was increased, indicating increased activity of the exchanger (Supplementary Figures S9D,H). Integral Ca^2+^ flux through RyR and SERCA also increased with increased *G*_TRPC6_ (Supplementary Figure S10), which explains the increase in SR load in TRPC6-eGFP vs. eGFP cells (Figure 5F). Increased NCX and SERCA activity for increased TRPC6 expression explain the reduced *T_Decay_* in both the model and experiments (Figures 5E,6E). Since NCX is electrogenic, its increased activity is also likely a driver of the observed slight depolarization of *V*_m_. With increased *I_TRPC_*_6_ leading to elevated [Ca^2+^]_i_, NCX extrudes more Ca^2+^ (Supplementary Figure S9H), exchanging each Ca^2+^ ion for three Na^+^ ions and resulting in a net increase of positive charge inside the cell (Supplementary Figure S9C). Resting *V*_m_ was marginally increased by increased *G*_TRPC6_ (Supplementary Figures S9A,E). Thus resting *V*_m_ is closer to Na^+^ channel activation threshold, so less current is required to trigger an action potential.

### Contribution of TRPC6 to Mechanical Feedback Mechanisms and Hypertrophic Remodeling

Our study shows direct effects of increased TRPC6 expression on [Ca^2+^]_i_ transients in cardiomyocytes. It was suggested that increased diastolic [Ca^2+^]_i_ increases diastolic tension and lowers the extent of cell relaxation (Louch et al., 2012). Due to the strong relationship of [Ca^2+^]_i_ and force in cardiomyocytes, we anticipate that the TRPC6-associated increase of [Ca^2+^]_i_ increases contraction. Similar effects can be expected in stretched cells, since TRPC6 is thought to be activated through membrane stretch (Spassova et al., 2006). TRPC6 knockout reduced stretch-induced slow force response by abolishing the slow increase in [Ca^2+^]_i_ (Yamaguchi et al., 2017). Our study with increased expression of TRPC6 supports their findings that the protein contributes to effects of the Frank-Starling mechanism by transiently increasing Ca^2+^ in response to stretch, thereby increasing contraction.

Previous studies linked TRPC6 to hypertrophy and increased calcineurin-NFAT signaling (Kuwahara et al., 2006; Onohara et al., 2006; Wu et al., 2010). NFAT was proposed to be a signal integrator of cumulative [Ca^2+^]_i_ load (Hannanta-Anan and Chow, 2016). It is thought that the role of TRPC6 in hypertrophic remodeling is through sustained and cumulative [Ca^2+^]_i_ increase that is sufficient to activate the calcineurin-NFAT pathway. A positive feedback in which Ca^2+^ influx through sarcolemmal TRPC6 channels activates calcineurin-NFAT leading to upregulation of TRPC6 and increased Ca^2+^ influx was suggested (Kuwahara et al., 2006). Our studies characterized the effects of upregulation of TRPC6 expression on [Ca^2+^]_i_, and we explain the effects by increased Ca^2+^ influx and SR load. Reduction of TRPC6 expression and/or activity is thus promising in disrupting the positive feedback loop as possible treatment for hypertrophic remodeling. Modulation of TRPC6 activity through pharmacological blockers is an active area of research (Bon and Beech, 2013). High-affinity potent inhibitors have been reported for TRPC6 including recent discoveries for orally available inhibitors (Washburn et al., 2013; Motoyama et al., 2018; Tang et al., 2018; Lin et al., 2019).

### Limitations

We note several limitations related to our studies. We did not find differences of our TRPC6 functional measures between NRVMs infected with shRNA-TRPC6-eGFP and eGFP (Figure 2, Supplementary Figure S8). While our immunofluorescence images suggest reduced expression of TRPC6 in shRNA-TRPC6 compared to eGFP (Supplementary Figure S5D), the reduction of TRPC6 expression is small. It is possible is that our shRNA-TRPC6-eGFP construct, originally designed to silence human TRPC6, is not efficiently reducing expression of TRPC6 in NRVMs. However, even a more pronounced reduction is unlikely to result in functional differences due to marginal native TRPC6 expression. Our finding of marginal TRPC6 expression is consistent with previously reported gene expression in NRVMs (dataset GSE83228), which revealed that expression of this TRPC6 gene is small vs. Cav1.2 (27x larger) and RyR2 expression (70x larger) (Stratton et al., 2016). Also, Jiang et al. suggested that TRPC6 protein expression is small during postnatal development and adulthood vs. expression at fetal stages (Jiang et al., 2014).

Limitations of the applied mathematical model are discussed in (Korhonen et al., 2009). Most importantly, NRVMs are known to exhibit variable phenotypes, especially in different culture conditions and during development. The model was built from data from several different laboratories where culture conditions were presumably heterogeneous and different to the conditions in our laboratory. The model is based on data from cells cultured 3–5 days whenever possible to minimize age-related variance, which aligns with our duration of culture. A major difference is the temperature at which experiments were performed. Temperature strongly affects cell function. For instance, Ca^2+^ release from the SR of rat cardiomyocytes is highly temperature dependent (Fu et al., 2005). The model is for NRVMs at 32°C, but our experiments were performed at room temperature to allow us to control the pacing rate. The temperature difference causes alterations of ion transport, channel gating and ion concentrations, including [Ca^2+^]_i_. For this reason, we only used the model for qualitative comparison of trends rather than actual measurements.

## Data Availability Statement

The datasets generated for this study are available on request to the corresponding author.

## Ethics Statement

The animal study was reviewed and approved by IACUC, University of Utah.

## Author Contributions

AA and FS designed the study. AA, FS, and MS drafted the manuscript. AS and MS acquired and analyzed image data. MS implemented the modeling and analyzed simulation data. CH isolated and infected cells. AA and CH performed western blotting. All authors critically revised the manuscript and approved the version to be published.

### Conflict of Interest

The authors declare that the research was conducted in the absence of any commercial or financial relationships that could be construed as a potential conflict of interest.
