# Supplementary material for: Modulation of Calcium Transients in Cardiomyocytes by Transient Receptor Potential Canonical 6 Channels
Source: Front Physiol. 2020 Feb 14;11:44. doi: 10.3389/fphys.2020.00044 (PMC7033547; doi:10.3389/fphys.2020.00044)
Supplement: Supplementary file 1 [file Data_Sheet_1.pdf]

SUPPLEMENTAL MATERIAL

Supplemental Methods

List of antibodies

Anti-TRPC6 antibodies (LS-C19628 and LS-B611, LifeSpan Bioscience, Seattle, WA, USA; AB105845, Abcam, Cambridge, MA, USA; PA-1754, Boster Bio, Pleasanton, CA, USA); DAPI (D3571, ThermoFisher, Grand Island, NY, USA); Donkey Anti-Rabbit IgG Alexa Fluor 647 (A31573, ThermoFisher); Donkey Anti-Rabbit IgG CF 568 (20098, Biotium, Fermont, CA, USA); GFP-Booster Alexa Fluor 647 (gb2AF647, Chromotek, Planegg-Martinsried, Germany); Precision Protein StrepTactin-HRP (1610380, Bio-Rad, Herculus, CA, USA); anti-eGFP (AB6556, Abcam); Goat Anti-Rabbit IgG-HRP (AB97069, Abcam); Goat Anti-Mouse IgG-HRP (AB97040, Abcam)

Anti-TRPC6 fluorescence measurements

Average intensities of LS-C19628 associated fluorescence were measured in confocal microscopic images from NRVMs infected with eGFP (n_{images}=2, n_{ROIs}=10, n_{cells}=8), TRPC6-eGFP (n_{images}=2, n_{ROIs}=5, n_{cells}=3), and shRNA-TRPC6-eGFP (n_{images}=2, n_{ROIs}=10, n_{cells}=10). Images were acquired as described in section 2.4. Image selection was limited to images acquired on the same day and cells positive for GFP. Mean fluorescence in regions of interest was determined using Fiji (Schindelin et al., 2012). Comparison of experimental data was performed using a one-way analysis of variables (ANOVA).

Supplemental Results

Table S1. Evaluation of anti-TRPC6 antibodies in NRVMs. For western blot and imaging, the protocols described in section 2.2 and 2.4, respectively, were applied. IF: Immunofluorescence

Catalog #	Immunogen	Listed Reactivity	Results
AB105845	Synthetic peptide for region near the C terminal of Human TRPC6	Human, chimpanzee	Western blot shows native and shifted band. IF shows no colocalization with TRPC6-eGFP.
LS-C19628	14 amino acid synthetic peptide for region (within amino acids 50-100) near N-terminus of human TRPC6.	Human, mouse	Western blot detects TRPC6-eGFP, but not native rat TRPC6. IF shows colocalization with TRPC6-eGFP, and marginal signals in eGFP and shRNA-TRPC6 cells.
LS-B611	14 amino acid synthetic peptide for region near C-terminus of human TRPC6. Located within the last 50 amino acids of TRPC6	Human, mouse, rat	Western blot shows native band, but no shifted band. IF does not show colocalization with TRPC6-eGFP, but marginal signals in eGFP cells.
PA-1754	Synthetic peptide corresponding to sequence in the middle region (amino acids 246-265) of human TRPC6	Human, mouse, rat	Western blot did not show bands for native TRPC6 and TRPC6-eGFP. IF not tested.

References

Schindelin, J., Arganda-Carreras, I., Frise, E., Kaynig, V., Longair, M., Pietzsch, T., Preibisch, S., Rueden, C., Saalfeld, S., Schmid, B., Tinevez, J.Y., White, D.J., Hartenstein, V., Eliceiri, K., Tomancak, P., and Cardona, A. (2012). Fiji: an open-source platform for biological-image analysis. *Nat Methods* 9**,** 676-682.
