# Supplementary material for: Modulation of Calcium Transients in Cardiomyocytes by Transient Receptor Potential Canonical 6 Channels
Source: Front Physiol. 2020 Feb 14;11:44. doi: 10.3389/fphys.2020.00044 (PMC7033547; doi:10.3389/fphys.2020.00044)
Supplement: Supplementary file 2 [file Presentation_1.pdf]

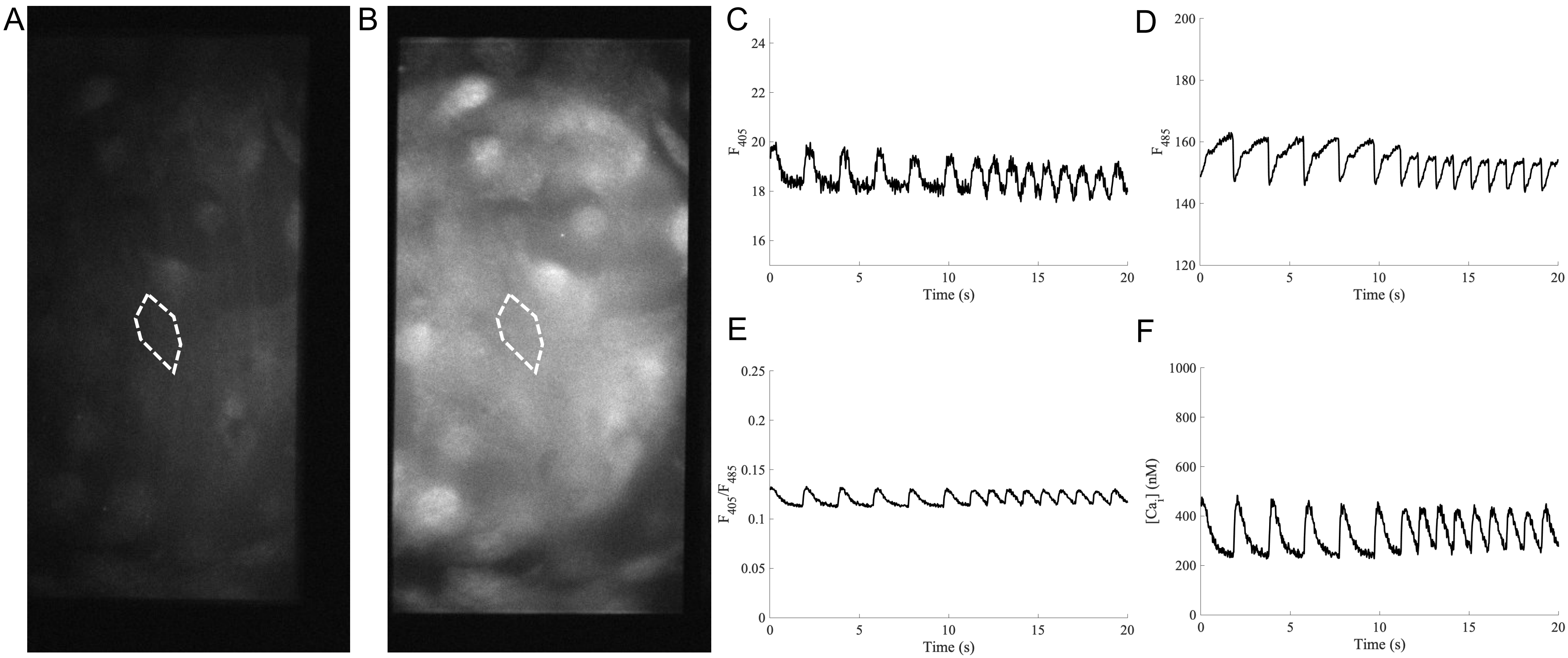

Figure S1. Image processing and analysis for pacing protocol. (A) F_{405} and (B) F_{485} image. (C) F_{405} transient extracted from single-cell ROI indicated by white dashed line in (A). (D) F_{485} transient from same ROI applied to (B). (E) F_{405}/F_{485} transient. (F) $[Ca^{2+}]_i$ calculated from (E) after calibration. All signals shown are after subtraction of camera background (F_{bg}) and cell autofluorescence (F_{cell}).

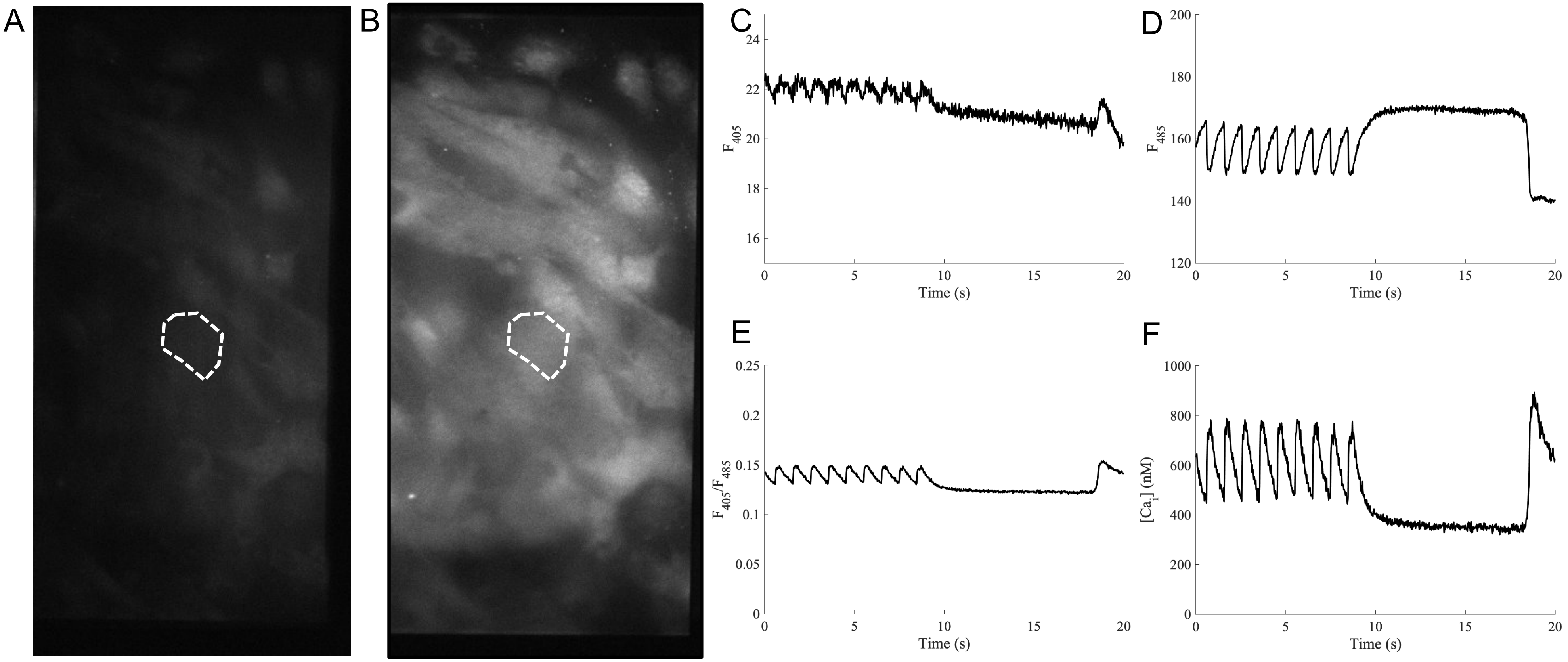

Figure S2. Image processing and analysis for protocol with caffeine application. (A) F_{405} and (B) F_{485} image. (C) F_{405} transient extracted from single-cell ROI indicated by white dashed line in (A). (D) F_{485} transient from same ROI applied to (B). (E) F_{405}/F_{485} transient. (F) $[Ca^{2+}]_i$ calculated from (E) after calibration. All signals shown are after subtraction of camera background (F_{bg}) and cell autofluorescence (F_{cell}).

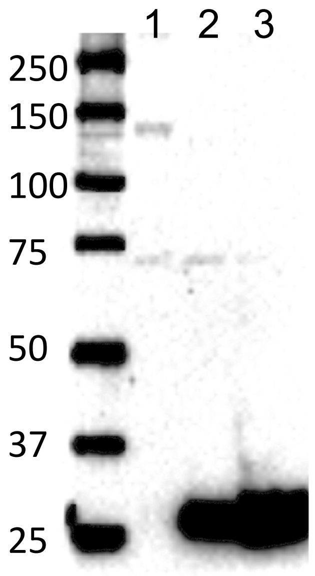

Figure S3. Contrast-enhanced western blot for Fig. 1E. Cells infected with TRPC6-eGFP (lane 1), eGFP (lane 2), and shRNA-TRPC6-eGFP (lane 3) were probed with anti-eGFP antibody at 10 second exposure. For TRPC6-eGFP cells, a band was detected at ~135 kDa, that was not present for eGFP and shRNA-TRPC6-eGFP cells. For eGFP and shRNA-TRPC6-eGFP cells, a band was detected at ~30 kDa, that was not present for TRPC6-eGFP cells.

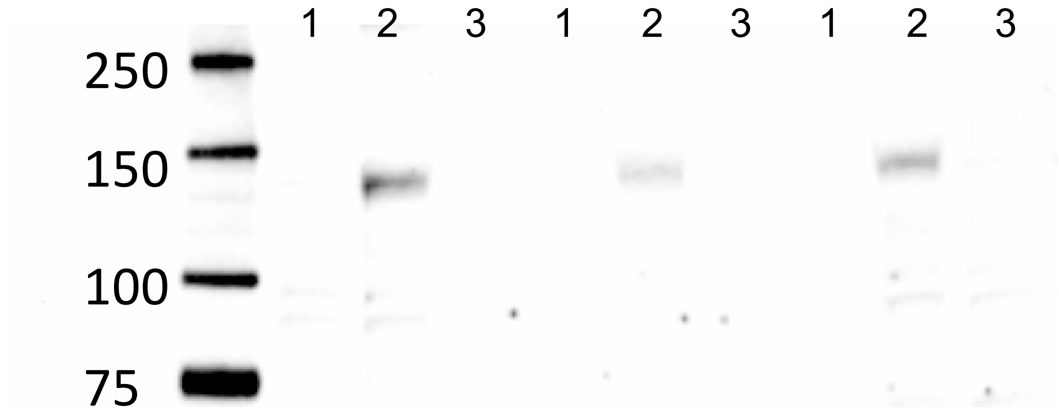

Figure S4. Western blot for anti-TRPC6 antibody LS-C19628 labeling NRVMs expressing eGFP (1), TRPC6-eGFP (2), and shRNA-TRPC6-eGFP (3). LS-C19628 identifies TRPC6-eGFP expression by a band ~135 kDa.

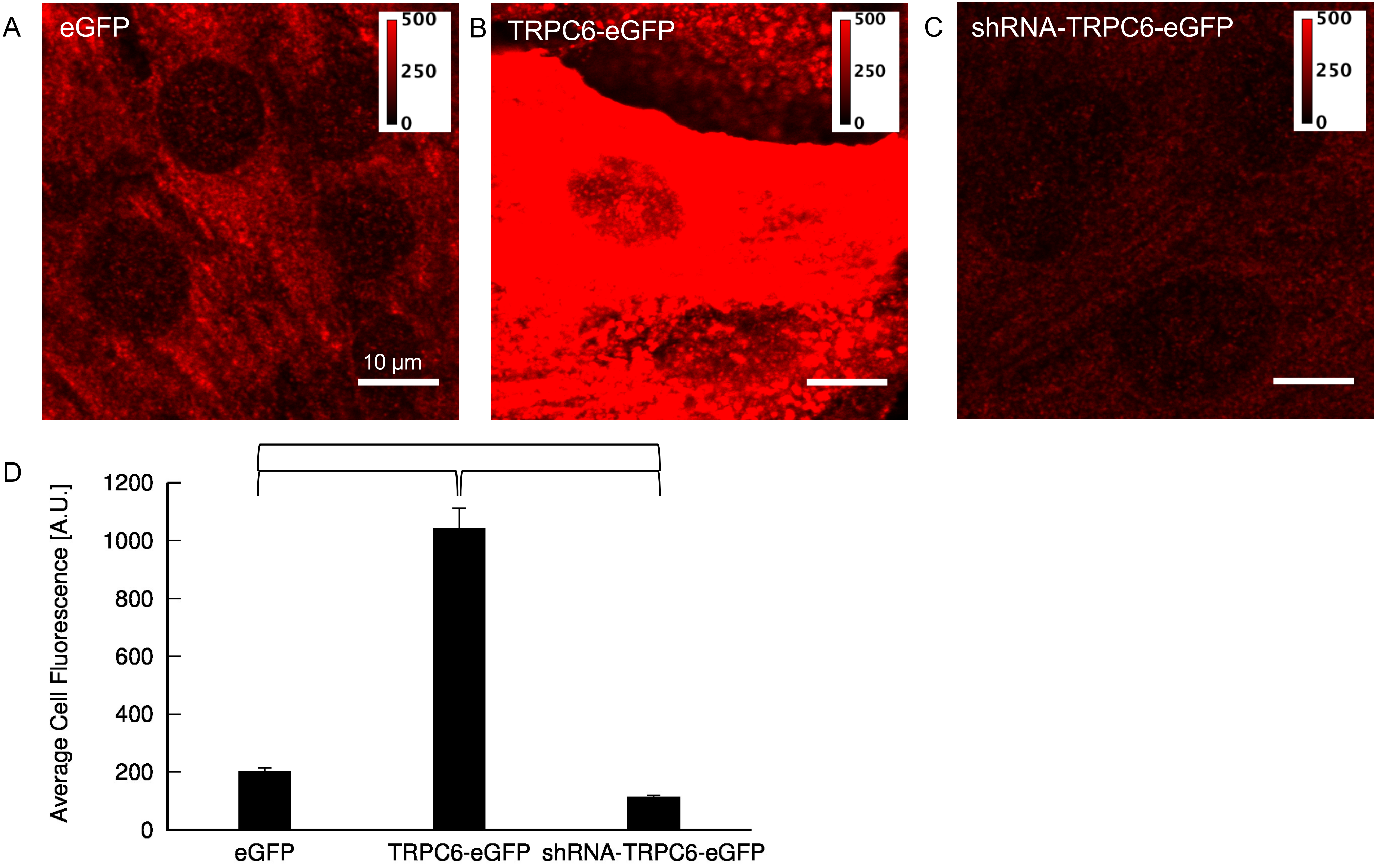

Figure S5. Brightness-adjusted images of anti-TRCP6 LS-C19628 labeling in (A) eGFP, (B) TRPC6-eGFP, and (C) shRNA-TRPC6-eGFP groups shown in Fig 2B, F, and J. (D) Fluorescence from anti-TRCP6 LS-C19628 labeling in NRVMs infected with eGFP, TRPC6-eGFP, and shRNA-TRPC6-eGFP.

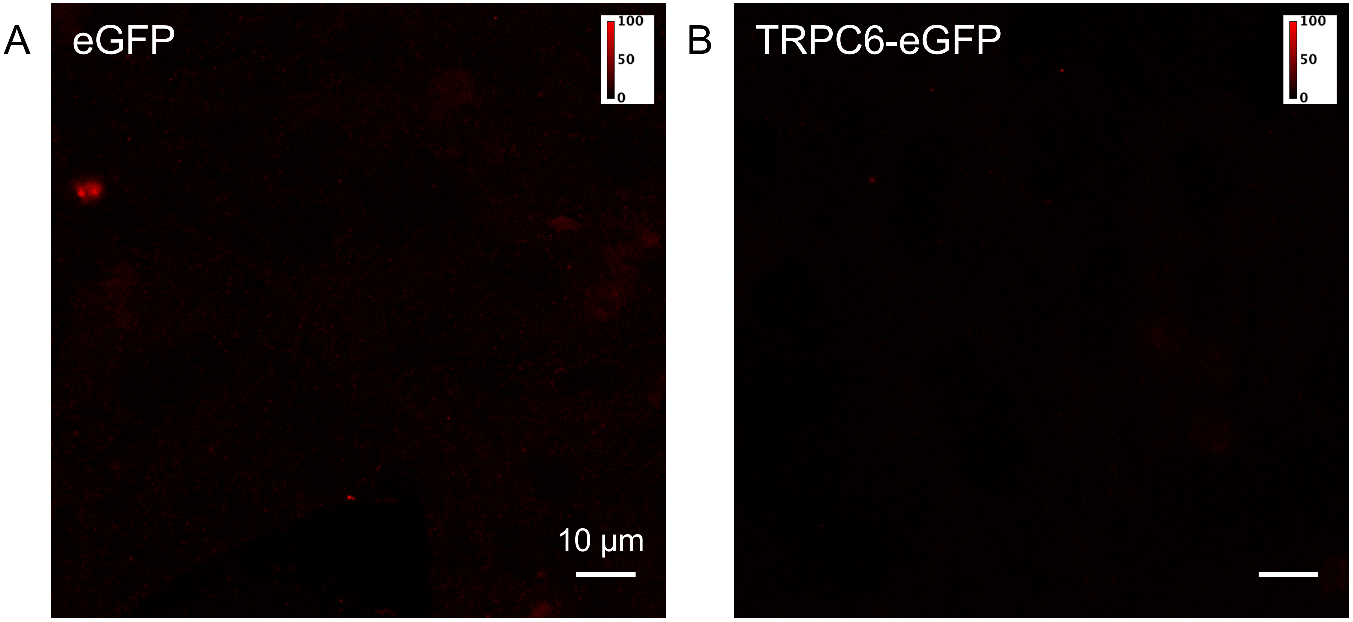

Figure S6. Images of secondary antibody only for (A) eGFP and (B) TRPC6-eGFP cells. Only marginal fluorescence was detected.

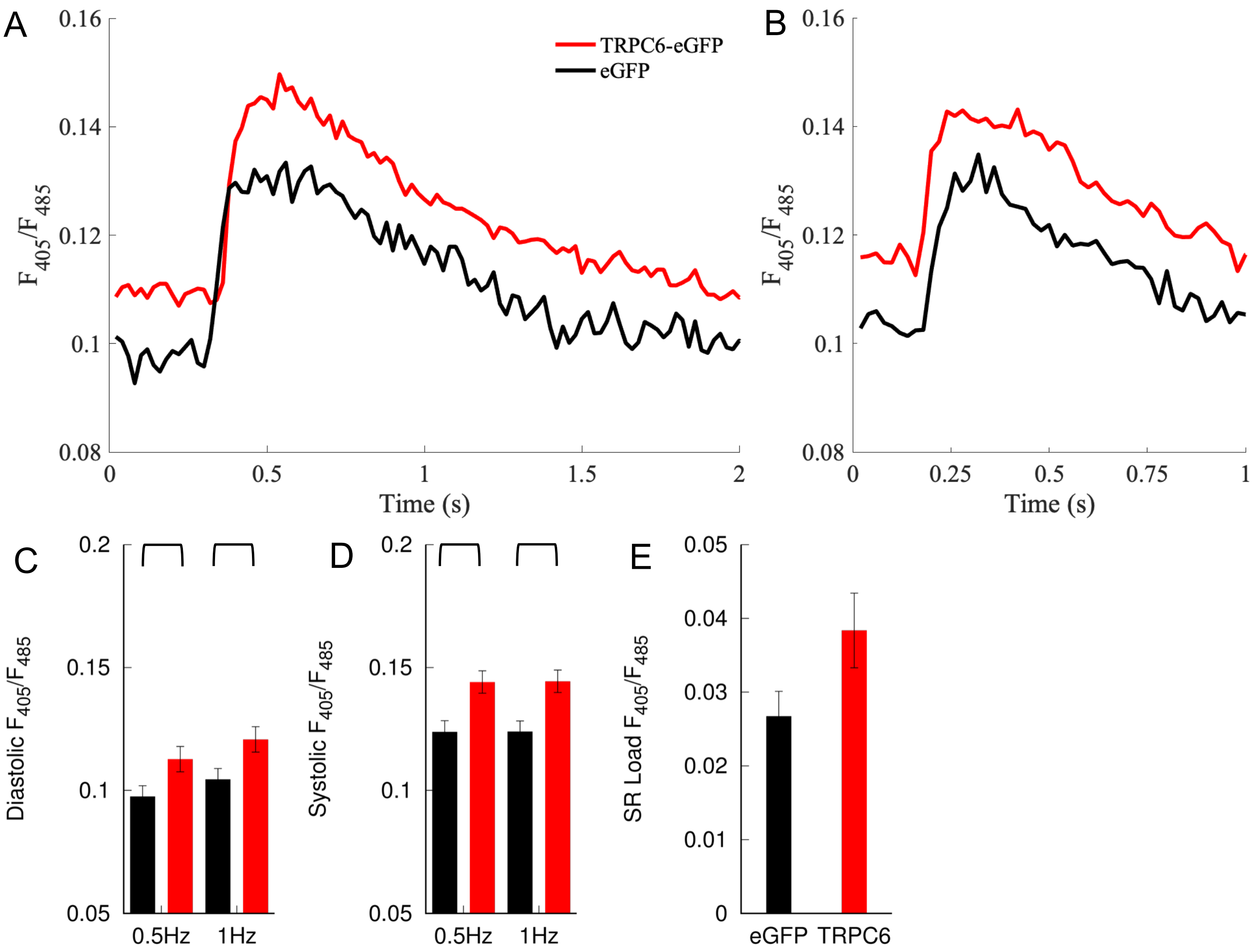

Figure S7. F_{405}/F_{485} for eGFP and TRPC6-eGFP. Example traces for each group with pacing at (A) 0.5 Hz and (B) 1 Hz. (C) Diastolic and (D) systolic F_{405}/F_{485} at each pacing rate. Both diastolic and systolic F_{405}/F_{485} increased at 0.5 and 1 Hz ($P<0.05$). (E) Amplitude of F_{405}/F_{485} for caffeine-induced SR load measurement.

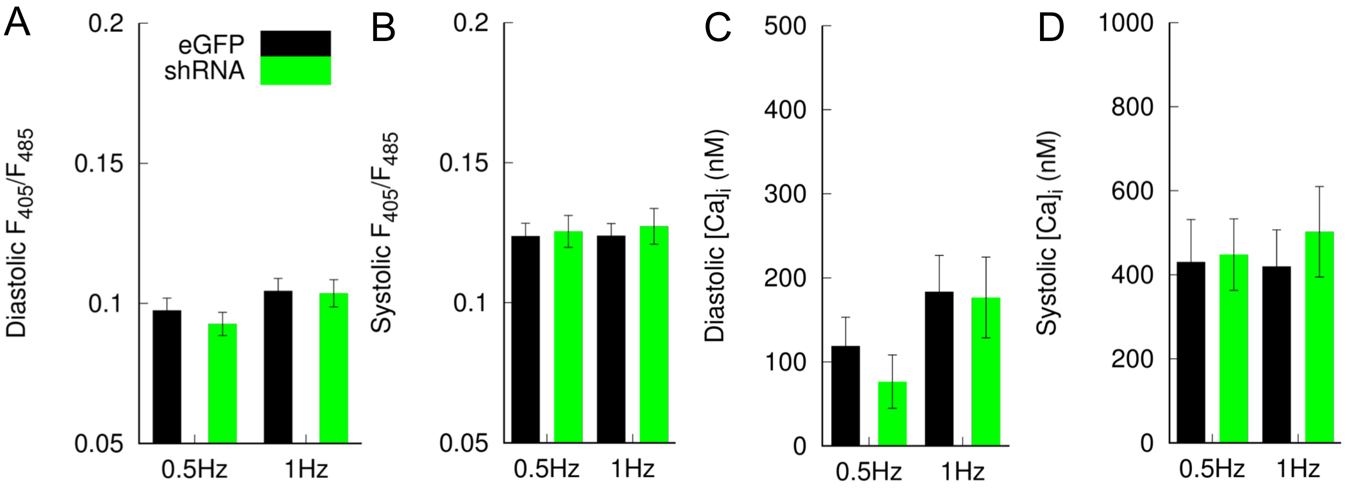

Fig S8. Assessment of native TRPC6 activity in NRVMs. Application of shRNA-TRPC6 to silence native TRPC6 did not yield significant differences in (A) diastolic or (B) systolic indo-1 ratios, at both 0.5 and 1 Hz pacing versus control ($P>0.05$). (C) Diastolic and (D) systolic $[Ca^{2+}]_i$ were not significantly different, suggesting marginal native TRPC6 contribution in NRVMs.

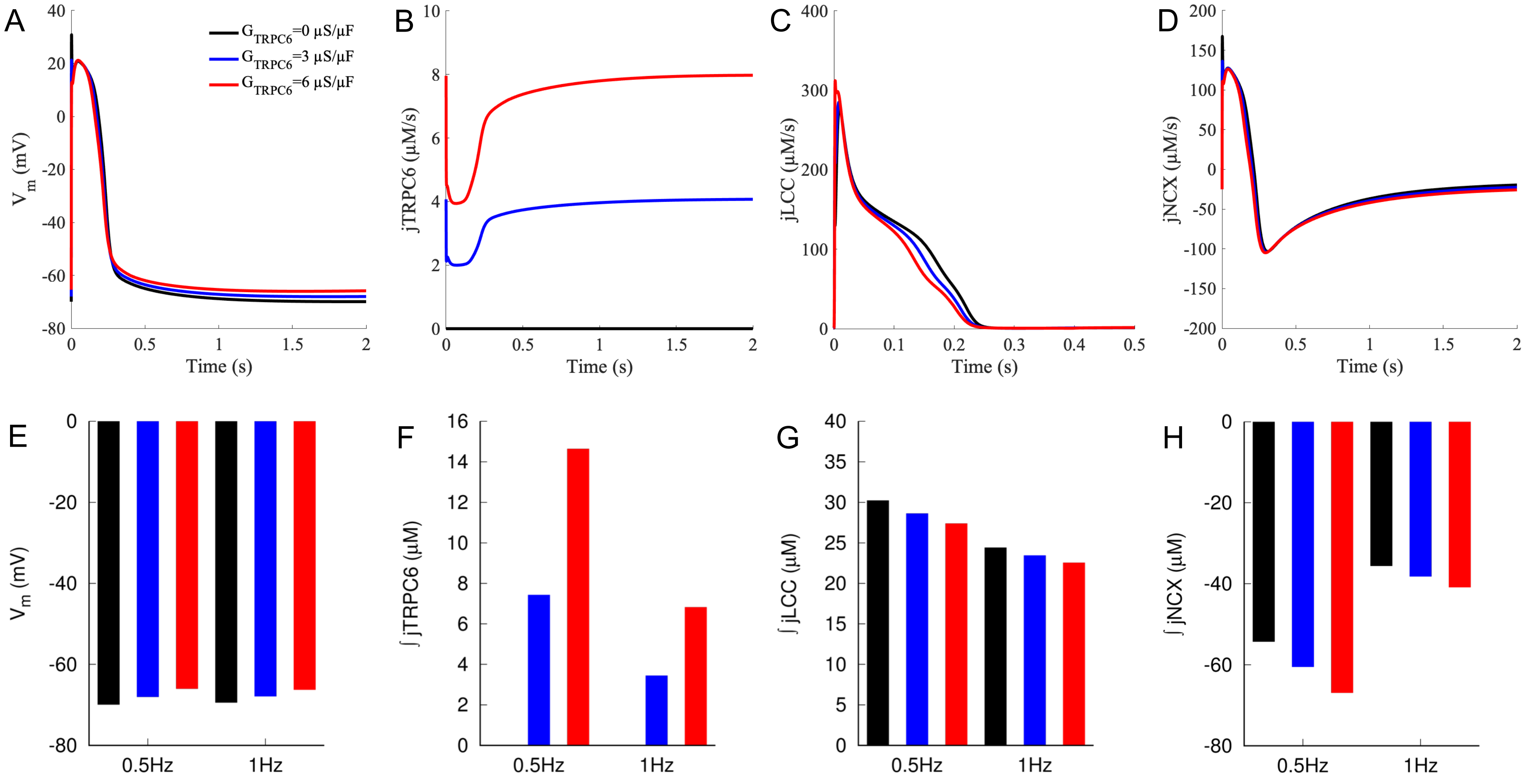

Fig S9. Simulation of NRVM electrophysiology and calcium signaling with TRPC6 modeled as sarcolemmal leak with G_{TRPC6} of 0, 3 and 6 µS/µF. (A) V_m in response to 0.5 Hz stimulation. Increased G_{TRPC6} decreased V_m. (B) Ca^{2+} flux through TRPC6. (C) L-type Ca^{2+} channel flux. Data are for first 0.5 s to show decrease of sarcolemmal channel activity with increased G_{TRPC6}. (D) Ca^{2+} flux through NCX channels. Negative values indicate forward mode of NCX. (E) Resting V_m increased for increasing G_{TRPC6} for 0.5 Hz and 1 Hz stimulation. (F) Ca^{2+} influx through TRPC6 is increased with increased G_{TRPC6}. (G) Ca^{2+} influx through LCC decreased with increased G_{TRPC6}. (H) Increased G_{TRPC6} led to an increased Ca^{2+} extrusion through NCX.

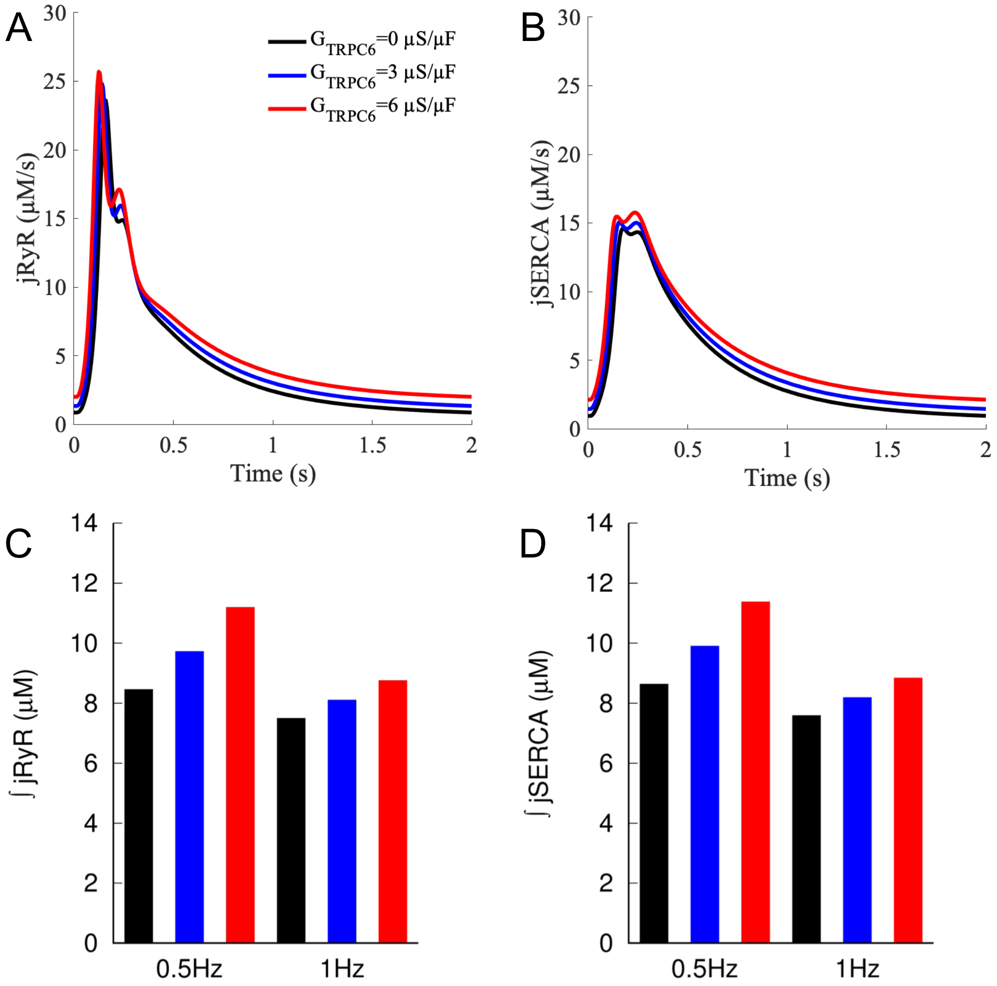

Fig S10. Simulation of SR Ca^{2+} dynamics in NRVM for G_{TRPC6} of 0, 3, and 6 μS/μF. (A) Ca^{2+} flux through RyRs for 0.5 Hz stimulation. RyR Ca^{2+} release is increased with increased G_{TRPC6}. (B) Ca^{2+} uptake through SERCA increases with increased G_{TRPC6}. (C) RyR release into the cytosol increased with increased G_{TRPC6}. (D) SERCA Ca^{2+} uptake increased with increased G_{TRPC6}.

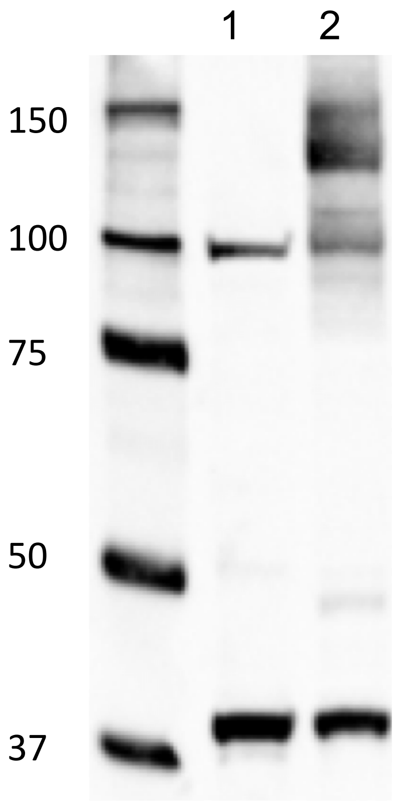

Figure S11. Western blot using anti-TRPC6 antibody AB-105845 for NRVMs infected with eGFP (lanes 1) and TRPC6-eGFP (lanes 2). AB-105845 identified marginal native TRPC6 expression by a weak band at ~100 kDa and strong TRPC6-eGFP expression by a band at ~135 kDa.
